# Frail older adults with minor fractures show lower health-related quality of life (SF-12) scores up to six months following emergency department discharge

**DOI:** 10.1186/s12955-016-0441-7

**Published:** 2016-03-08

**Authors:** Véronique Provencher, Marie-Josée Sirois, Marcel Émond, Jeffrey J. Perry, Raoul Daoust, Jacques S. Lee, Lauren E. Griffith, Brice Lionel Batomen Kuimi, Litz Rony Despeignes, Laura Wilding, Nadine Allain-Boulé, Johan Lebon, On behalf of the Canadian Emergency Team Initiative on Mobility in Aging

**Affiliations:** Université de Sherbrooke, and Centre de recherche sur le vieillissement, Sherbrooke, QC Canada; Université Laval, and Centre de Recherche du CHU de Québec, Quebec, QC Canada; University of Ottawa, and Ottawa Hospital Research Institute, Ottawa, ON Canada; Université de Montréal, and Research Center, Hôpital du Sacré-Cœur de Montréal , Montréal, QC Canada; University of Toronto, and Sunnybrook Health Science Center, Toronto, ON Canada; McMaster University, Hamilton, ON Canada

**Keywords:** Frail elderly, Health-related quality of life, Minor fractures, Emergency department, Geriatric assessment

## Abstract

**Background:**

Minor fractures (e.g. wrist, ankle) are risk factors for lower physical health-related quality of life (HRQoL) in seniors. Recent studies found that measures of frailty were associated with decreased physical and mental HRQoL in older people. As most people with minor fractures go to emergency departments (EDs) for treatment, measuring their frailty status in EDs may help stratify their level of HRQoL post-injury and provide them with appropriate health care and services after discharge. This study thus examines the HRQoL of seniors visiting EDs for minor fractures at 3 and 6 months after discharge, according to their frailty status.

**Methods:**

This prospective sub-study was conducted within the larger Canadian Emergency Team Initiative (CETI) cohort. Independent seniors (≥65 years) were recruited in 7 Canadian EDs after treatment for various minor fractures. Frailty status in the ED phase was assessed by the *Canadian Study of Health and Aging--Clinical Frailty Scale* (CSHA-CFS). The SF-12 questionnaire was completed at 3 and 6 months after ED discharge to ascertain HRQoL. Demographic and clinical data were collected. Linear mixed models were used to test for differences between frailty levels and HRQoL outcomes, controlling for confounding variables and repeated measures over time.

**Results:**

The sample comprised 334 participants with minor fractures. Prevalence of frailty was as follows: 56.6 % *very fit-well*; 32.3 % *well with treated comorbidities-apparently vulnerable*; and 11.1 % *mildly-moderately frail*. After adjusting for confounding variables, the frailest group showed significantly lower mean HRQoL scores than the fittest group on the physical scale at 3 months (49.3 ± 3.7 *vs* 60.9 ± 2.0) and 6 months (48.7 ± 3.8 *vs* 61.1 ± 1.8), as well as on the mental scale at 3 months (59.5 ± 4.4 *vs* 69.6 ± 1.9). Analyses exploring differences in proportion of patients with HRQoL < 50/100 between the three groups produced similar results.

**Conclusions:**

Older adults with minor fractures who were frail had lower physical and mental HRQoL scores at 3 and 6 months after ED discharge than their fittest counterparts. Measuring the frailty status of older adults who suffered a minor fracture in ED might help clinical decision-making at the time of discharge by providing them with appropriate health care and services to improve their HRQoL in the following months.

## Background

Older adults account for an increasing proportion of visits to North American emergency departments (EDs) [1, 2]. Fractures are the most common reason (41 %) for injurious fall-related ED visits by seniors [3]. About half (49 %) of these fractures can be considered “minor” as they do not result in hospital admission [3]. As most seniors with injuries like minor fractures return home following their ED visit [4], it is important to ensure that they receive the appropriate healthcare to optimize their health-related quality of life (HRQoL) after ED discharge.

The concept of HRQoL can be defined as aspects of quality of life that are influenced by, or that can influence, one’s health status directly [5]. The Short Form-12 Health Survey (SF-12) is one of the most frequently used generic tools for assessing the physical and mental components of a person’s HRQoL [6]. A cross-sectional study done by Sanfélix et al. [7] revealed that the physical dimension of HRQoL, measured with the SF-12, was significantly lower in older adults having suffered a minor fracture (spine or wrist), compared to their uninjured counterparts. In one of the few studies to investigate the evolution of HRQoL in patients with a minor fracture (wrist) following ED discharge, Gonzalez et al. (2014) [8] found that the physical dimension of the SF-12 deteriorated up to 6 months after the injury, especially in older women. However, little is known about the characteristics of older adults who show poorer HRQoL following a minor fracture. It is thus essential to identify those seniors so that appropriate healthcare and community-based services may be provided following their ED visits.

One hypothesis is that older adults with minor fractures who demonstrate poorer HRQoL following ED visits are in a frail state. Frailty refers to a state of increased vulnerability to stressors, leading to higher risks for adverse outcomes such as falls and fractures [9], as well as disability, hospitalization and mortality. Recently, several cross-sectional studies [10–13] reported worse HRQoL measures in frail community-dwelling adults than in non-frail ones. This association was also found in a recent prospective study by Bagshaw [14] with a sample of survivors of critical illness, among whom frail individuals showed lower physical and mental HRQoL in the year after discharge from an intensive care unit, compared to their non-frail counterparts. Based on these previous studies conducted in other settings and on other populations, we expected that measuring the frailty status of older adults who suffered a minor fracture before ED discharge might help to stratify their level of HRQoL post-injury and improve clinical decision-making at discharge by implementing a healthcare approach tailored to the specific needs of these patients. Targeting these patients in ED may thus help to provide them with appropriate health care and services upon returning home, as some community-based interventions [15] and virtual ward program [16] recently showed promising results to improve HRQoL in frail older adults. To our knowledge, no previous study has documented the prospective relationship between frailty and HRQoL among ED patients treated for minor fractures. This study thus aimed to compare the HRQoL measures of seniors visiting EDs for minor fractures, according to their frailty status.

## Methods

### Study design and population

This prospective sub-study was conducted within the larger Canadian Emergency Team Initiative (CETI) research program on mobility and aging, aimed at improving ED care for independent seniors with minor injuries (http://www.cha.quebec.qc.ca/ceti/). Data for the current study were collected over 2 years between March 2011 and March 2013 in 7 EDs in 5 Canadian cities (Quebec, Montreal, Ottawa, Toronto and Hamilton). Clinicians and/or research assistants identified and screened consecutive potential participants (24/7). Participants were included in the study if they: 1) were aged 65 years or older; 2) presented to the ED with the chief complaint being a minor fracture (bone lesions of lower or upper limbs, head, chest or spine); 3) were independent in Basic Activities of Daily Living (BADL) in the 4 weeks preceding the injury, based on the Older Americans Resources and Services (OARS) [17] questionnaire; and 4) were sent home. Patients were excluded if they: 1) presented with significant injuries leading to hospitalization; 2) were unable to give verbal consent or attend follow-up evaluations; 3) resided in long-term care facilities pre-trauma; or 4) were unable to communicate in French or English.

### Data collection procedure

Demographic and clinical data were collected by trained research assistants during initial evaluation and follow-ups (at 3 and 6 months), except for the medical information pertaining to the fractures, which was documented by the treating physicians during the ED visit. For about 30 % of the sample (randomly selected participants), data pertaining to HRQoL were collected through in-person assessments at 3 and 6 months at the different centers. For the remaining participants, phone interviews were conducted. The frailty status assessments for all participants were performed in-person by the treating physicians during the initial ED visit.

All research assistants received training on the tools and questionnaires. Data collection quality and standardization were maintained through regular telephone and in-person team meetings.

The study was approved by the research ethics board of each recruiting site and written informed consent was obtained from study participants.

### Variables and outcomes

Frailty status was assessed by the *Canadian Study of Health and Aging--Clinical Frailty Scale (CSHA-CFS)* [18]. This tool is based on the judgment of clinicians and has been validated in a population-based study of Canadian seniors. It classifies older adults as *very fit* (level 1)*, well* (level 2), *well with treated comorbidities* (level 3), *apparently vulnerable* (level 4), *mildly frail* (level 5), *moderately frail* (level 6) or *severely frail* (level 7). In the present study, participants were classified according to three frailty status: *very fit-well* (level 1–2); *well with treated comorbidities-apparently vulnerable* (level 3–4); and *mildly to severely frail* (level 5–7). To make this article easier to read, these three groups are referred to as: 1) *fit*, 2) *vulnerable,* and 3) *frail*.

Health-related quality of life was determined using the SF-12 [19]. This tool has been shown to be a reliable and valid measure of health status in older adults living independently [20]. The SF-12 is a brief, widely used questionnaire, which consists of 12 questions covering 8 health domains: general health, mental health, vitality, social functioning, role limitation due to physical health problems, role limitation due to emotional problems, bodily pain limiting usual activities, and physical functioning. These domains can be summarized in a Physical Component Summary scale (PCS) and Mental Component Summary scale (MCS), ranging from 0 (worst) to 100 (best) score. A score of 50 or more indicates positive self-rated health, while a score below 50 indicates a negative perception [8].

Potential confounding demographic and clinical variables considered in this study were measured at baseline. Age, gender, schooling (<12 years), living alone (without help), ED visits in the last 3 months, falls in the last 3 months, number of comorbidities (categorized in 0–4 and 5–13), number of outings/week (<5) and occasional use of a walking aid (yes/no) were collected through self-reported interview. Types, locations, and severity of each fracture as well as co-occurring injuries were coded by trained professional according to the 2005 revision of the *Abbreviated Injury Scale* (AIS) [21]. AIS severity codes from 1(minor) to 6 (maximum) were used to compute the aggregated Injury Severity Scores (ISS) which ranges from 1 (minor) to 75 (unsurvivable) [22, 23]. Self-reported pain intensity was measured by *0 to 10 Visual Analog Scale* [24]. Basic (BADL) and instrumental activities of daily living (IADL) scores were measured by the *Older American Resources Scale (OARS)* [25]. The OARS has been widely validated and shows good test-retest reliability [26]. Fall efficacy (fear of falling) was measured by the *Short Falls Efficacy Scale-International (short FES-I)* [27] which has excellent test-retest reliability. Cognitive status was measured by the *Montreal Cognitive Assessment (MoCA)* [28] or the modified *Telephone Interview for Cognitive Status (TICS-m)* [29]. The MoCA is a brief, reliable and sensitive to decline in cognitive function [28]. The TICS-m is useful an economical alternative to standard in-person screening for population studies [29, 30]. Social support was measured by the *Social support index (SSI) from Québec Health Surveys* [31]. It measures both quantity and satisfaction with available support. It has been validated in the general population of the province of Québec in both French and English and norms are available (a higher score indicates lower support) [31]. All the variables mentioned above were selected based on their clinical relevance and correlation with the main variables [8, 32]. Cut-offs for potential covariates were published clinical cut-offs when available (e.g. FES-I, MoCA, TICS-m, SSI) or those found out to be the most predictive of post-injury functional decline in a recent study (e.g. education, number of outings, pain) [33].

### Statistical analysis

Descriptive analyses were performed to characterize the sample. Simple linear mixed models were used to explore differences in HRQoL between the three groups (frail, vulnerable, fit) at 3 and 6 months. This type of analysis accounts for within-subject correlation among repeated measures.

Multivariate linear mixed models using a stepwise backward selection strategy were used to identify potential confounding variables. Only significant variables (*p* < 0.10) pertaining to demographics and health that impacted on the frailty coefficient (change over 10 %) were kept in the final adjusted models, also accounting for repeated measures over time. For the PCS, the variables retained were schooling, number of comorbidities, occasional use of a walking aid, baseline IADL score, and number of outings/week. Age and cognitive status were also found to be significant for the MCS. Linear mixed models were then used to test for differences between frailty levels and HRQoL outcomes, controlling for confounding variables and repeated measures over time. A paired-t test was also done to detect significant differences in time measures within each group on the PCS and MCS. Finally, the relative proportions (RP) of patients with a HRQoL < 50/100 between the three groups (frail, vulnerable, fit) at 3 months and 6 months were calculated. These analyses were conducted using a general estimating equations approach. All analyses were performed with PROC MIXED and GENMOD using SAS 9.4 (SAS Institute Inc., Cary, NC).

## Results

A total of 428 participants who had suffered a minor fracture were initially enrolled in the study. Of those, 334 completed the HRQoL assessment at least one of the two time points (3 and 6 months). Baseline characteristics of participants and dropouts are detailed in Appendix. Mean age of participants was 76 ± 7.3 years. Most fractures were caused by a fall from own or greater height (74.7 %). Main reason for dropouts was that they could not be reached within the allowed timeframe (19.4 %). No significant differences were found between participants and those lost to follow-up (3 and 6 months), except for schooling; older adults lost to follow-up had less schooling than participants. Loss to follow-up (fit: 48.9 %; vulnerable: 38.9 %; frail: 12.2 %) did not differ significantly between the three groups (*p* = 0.42). On the SF-12, average mental scores (3 months: 67.3 ± 19.0; 6 months: 69.3 ± 16.7) and physical scores (3 months: 66.1 ± 22.6; 6 months: 65.3 ± 20.8) remained quite stable between time measures.

Participants’ characteristics according to their frailty status are shown in Table 1. The proportions of participants in the 3 groups were as follows: 56.6 % were *very fit-well* (level 1–2); 32.3 % were *well with treated comorbidities or apparently vulnerable* (level 3–4); and 11.1 % were *mildly to moderately frail* (level 5–6). Because of the inclusion criteria, no participants were classified as *severely frail* (level 7) on the *CSHA Clinical Frailty Scale.* Participants in the frailest group were older, had more comorbidities, showed less fall efficacy and presented with more IADL disabilities than the other two groups. Also, more participants in that group reported occasionally using a walking aid. The vulnerable group had less schooling. The fittest group demonstrated less cognitive impairment, had higher IADL scores and went out more often than the other two groups.

**Table 1:** Participants’ characteristics according to their frailty status

	Very fit--well (*N* = 189)	Well + comorbid--Apparently vulnerable (*N* = 108)	Mildly frail--Moderately frail (*N* = 37)	*p*-value*
Age				
65-74	98 (51.9)	42 (38.9)	8 (21.6)	0.0006
75-85	77 (40.7)	50 (46.3)	19 (51.4)	
85+	14 (7.4)	16 (14.8)	10 (27.0)	
Male	45 (23.3)	24 (22.4)	9 (24.3)	1.0
Schooling				
Primary	14 (7.6)	17 (16.0)	4 (11.1)	0.0004
Secondary	55 (29.7)	47 (44.3)	11 (30.6)	
College	40 (21.6)	23 (21.7)	3 (8.3)	
University	76 (41.1)	19 (17.9)	18 (50.0)	
Number of comorbidities				
0–4	125 (66.1)	59 (54.6)	14 (38.9)	0.004
5–13	64 (33.9)	49 (45.4)	22 (61.1)	
Living alone, without help	66 (35.1)	36 (33.3)	9 (24.3)	0.4
Social Support Index (SSI > 63/100)	159 (84.6)	83 (78.3)	24 (68.6)	0.06
ED visits in the last 3 months	21 (11.2)	17 (15.9)	4 (11.8)	0.5
Falls in the last 3 months	31 (16.4)	24 (22.2)	7 (20.0)	0.5
Numbers of outings/week < 5	150 (82.9)	69 (63.9)	18 (56.3)	0.0001
Occasional use of a walking aid	17 (9.1)	25 (23.2)	17 (47.2)	<0.0001
Baseline BADL score (14/14)	137 (72.5)	98 (90.7)	29 (78.4)	<0.0001
Baseline IADL score (<14)	137 (72.5)	51 (47.2)	15 (40.5)	<0.0001
Injury mechanism^a^				
- Fall from own height	134 (72.0)	80 (75.5)	31 (86.1)	0.5
- Fall from greater height	24 (12.9)	16 (15.1)	4 (11.1)	
- Motor vehicle accident	5 (2.7)	3 (2.8)	0 (0.0)	
- Others	23 (12.4)	7 (6.6)	1 (2.8)	
Location of fracture (injured body region)^a^				
Head	9 (4.8)	6 (5.6)	3 (8.1)	0.6
Spine	5 (2.7)	3 (2.8)	3 (8.1)	
Upper limb	104 (55.0)	53 (49.1)	22 (59.5)	
Thorax	27 (14.3)	17 (15.7)	5 (13.5)	
Lower limb	37 (19.6)	25 (23.2)	4 (10.8)	
Multiple	7 (3.7)	4 (3.7)	0 (0.0)	
Description of injury				
Fracture only	133 (70.4)	61 (56.5)	28 (75.7)	0.02
With co-occurring injuries^b^	56 (29.6)	47 (43.5)	9 (24.3)	
Tear	1 (0.5)	1 (0.9)	0	
Abrasion	9 (4.8)	12 (11.1)	0	
Luxation	1 (0.5)	0	0	
Laceration	10 (5.3)	16 (14.8)	0	
Contusion	26 (13.8)	25 (23.2)	3 (8.1)	
Sprain	5 (2.7)	4 (3.7)	2 (5.4)	
Avulsion	1 (0.5)	1 (0.9)	1 (2.7)	
Effusion	1 (0.5)	0	0	
Traumatic brain injury	16 (8.5)	14 (13)	5 (13.5)	
Injury Severity Score (ISS) (/75)				
1–2	22 (11.6)	10 (9.3)	3 (6.9)	0.1
3–4	141 (74.6)	70 (64.8)	27 (62.7)	
5 +	26 (13.8)	28 (25.9)	7 (18.9)	
Pain (Visual Analog Scale ≥7/10)	20 (10.7)	14 (13.0)	6 (16.2)	0.6
Short Falls Efficacy Scale-International (<9.8)	120 (63.5)	56 (51.9)	14 (38.9)	0.01
Cognitive status (MoCA < 23/30 or TICS-modified ≤ 31/50)	36 (19.7)	37 (35.2)	10 (28.6)	0.01

*Chi-Square ^a^Fisher test ^b^Not mutually exclusive categories

Before adjusting for confounding variables, the PCS and MCS mean scores differed significantly (*p* = <0.01) between the 3 groups (frail, vulnerable, fit) at 3 and 6 months: frail participants showed lower unadjusted PCS and MCS mean scores at all time points than the other two groups (see Table 2). After adjusting for confounding variables, the frailest group showed lower PCS scores (*p* = <0.01) than the fittest group at 3 months and 6 months, as well as on the MCS (*p* = 0.03) at 3 months. The frailest group also scored lower on the PCS (*p* = <0.01) than the vulnerable group at 3 months, while the vulnerable group scored lower (*p* = 0.01) than the fittest group on the MCS (See Table 3). Other analyses produced similar results: the frailest group showed higher proportions of HRQoL < 50/100 (indicating negative self-rated health) than the fittest group on the PCS at 3 months (relative proportion [RP]: 1.76, *p* = 0.02) and 6 months (RP: 2.40, *p* < 0.01), as well as on the MCS at 3 months (RP: 2.40, *p* = 0.01) and 6 months (RP: 2.56, *p* = 0.01). The frailest group also show higher proportions of HRQoL < 50 than the vulnerable group on the PCS at 3 months (RP: 2.15, *p* = 0.01), as well as on the MCS at 6 months (RP: 2.85, *p* = 0.01) (see Table 4).

**Table 2:** Unadjusted HRQoL mean scores^a^, according to the frailty groups at 3 months and 6 months

	Mean (95 % CI)	Standard of Error	*P* value
Physical Component Summary SF-12 score^b^			
3 months^c^			
- Very fit--well	69.6 (66.2 72.7)	21.9	
- Well + comorbid--Apparently vulnerable	65.6(61.4 69.9)	21.4	
- Mildly frail--Moderately frail	51.9 (43.9 59.9)	24.1	<0.001
6 months			
- Very fit--well	70.2 (67.3 73.1)	17.8	
- Well + comorbid--Apparently vulnerable	61.6 (56.7 66.4)	22.1	
- Mildly frail--Moderately frail	50.5 (41.7–59.3)	23.1	<0.001
Mental Component Summary SF-12 score^d^			
3 months			
- Very fit--well	70.6 (68.1 73.1)	16.5	
- Well + comorbid--Apparently vulnerable	64.5 (60.5–68.5)	20.3	
- Mildly frail--Moderately frail	59.2 (51.7 66.8)	22.7	<0.001
6 months			
- Very fit--well	71.0 (68.773.2)		
- Well + comorbid--Apparently vulnerable	69.7 (66.0–73.5)		
- Mildly frail--Moderately frail	59.6 (50.4–68.8)		0.003

^a^obtained by linear mixed model

^b^Adjustments based on schooling, comorbidities, use of walking aid, baseline IADL score, and number of outings per week

^c^Very fit—well (*n* = 173); Well + comorbid--Apparently vulnerable (*n* = 101); Mildly frail--Moderately frail (*n* = 37)

^d^Adjustments based on age, schooling, comorbidities, use of walking aid, baseline IADL score, cognitive status, and number of outings per week

**Table 3:** Adjusted HRQoL mean scores^a^, according to the frailty groups at 3 months and 6 months

	Mean (95 % CI)	Standard of Error	P value
Physical Component Summary SF-12 score^b^			
3 months^c^			
- Very fit--well	60.9 (57.0–64.8)	2.0	
- Well + comorbid--Apparently vulnerable	62.4 (58.1–66.7)	2.2	0.6
- Mildly frail--Moderately frail	49.2 (42.1–56.5)	3.7	0.005
6 months			
- Very fit--well	61.1 (57.6–64.8)	1.8	
- Well + comorbid--Apparently vulnerable	57.5 (52.6–62.5)	2.5	0.2
- Mildly frail--Moderately frail	48.7 (41.2–56.2)	3.8	0.003
Mental Component Summary SF-12 score^d^			
3 months			
- Very fit--well	69.6 (65.8–73.4)	1.9	
- Well + comorbid--Apparently vulnerable	63.5 (59.2–67.7)	2.2	0.014
- Mildly frail--Moderately frail	59.5 (50.9–68.1)	4.4	0.032
6 months			
- Very fit--well	70.1 (66.5–73.7)	1.84	
- Well + comorbid--Apparently vulnerable	69.0 (65.2–72.9)	1.94	0.6
- Mildly frail--Moderately frail	60.8 (51.1 − 70.6)	4.96	0.08

^a^obtained by linear mixed model

^b^Adjustments based on schooling, comorbidities, use of walking aid, baseline IADL score, and number of outings per week

^c^Very fit—well (*n* = 173); Well + comorbid--Apparently vulnerable (*n* = 101); Mildly frail--Moderately frail (*n* = 37)

^d^Adjustments based on age, schooling, comorbidities, use of walking aid, baseline IADL score, cognitive status, and number of outings per week

**Table 4:** Adjusted proportions^a^ (score < 50), according to the frailty groups at 3 months and 6 months

	Proportion (95 % CI)	Standard of Error	*P* value
Physical Component Summary SF-12 score^b^			
3 months			
- Very fit--well	26.6 (19.1–36.9)	4.5	
- Well + comorbid--Apparently vulnerable	21.7 (14.9–31.7)	4.2	0.4
- Mildly frail--Moderately frail	46.7 (32.1–67.9)	8.9	0.02
6 months			
- Very fit--well	16.8 (10.9–25.9)	3.7	
- Well + comorbid--Apparently vulnerable	34.4 (24.8–47.7)	5.7	0.006
- Mildly frail--Moderately frail	40.3 (27.5–59.1)	7.9	0.003
Mental Component Summary SF-12 score^c^			
3 months			
- Very fit--well	12.4 (7.4–20.7)	3.3	
- Well + comorbid--Apparently vulnerable	18.9 (11.3–31.2)	4.9	0.2
- Mildly frail--Moderately frail	29.7 (16.6–53.4)	8.9	0.01
6 months			
- Very fit--well	10.1 (5.7–17.8)	2.9	
- Well + comorbid--Apparently vulnerable	9.1 (4.4–19.0)	3.4	0.8
- Mildly frail--Moderately frail	25.9 (14.1–47.5)	8.0	0.01

^a^obtained by sensitivity analyses were conducted using a general estimating equations approach

^b^Adjustments based on age, schooling, comorbidities, use of walking aid, baseline IADL score, and number of outings per week

^c^Adjustments based on schooling, income, comorbidities, use of walking aid, cognitive status, and fear of falling

Within each group, no significant changes were reported in mean SF-12 scores over time (3 vs 6 months) for the frailest and fittest groups. More variations in SF-12 scores were found in the vulnerable group: MCS scores increased (*p* < 0.01) between 3 and 6 months (from 63.5 to 69.0), while PCS scores decreased (*p* = 0.04) over the same interval (from 62.4 to 57.5). Based on Al Sayah et al. [32], this change in HRQoL scores can be considered clinically significant (difference ≥ 5 points).

## Discussion

Our study found that participants with minor fractures who were frail (but independent in BADL before the injury) had worse physical and mental HRQoL up to 6 months after ED discharge than their fittest counterparts. This conclusion was supported by the two kinds of analyses performed (e.g., based on difference in HRQoL mean and in proportion of patients with low HRQoL score) which strengthen the results of this study. Our results are consistent with those of a recent study by Bagshaw et al. [14], which found that frailty, measured with the same scale (*Clinical Frailty Scale*) and using the same cut-offs (>4), was associated with poorer mental and physical HRQoL one year after discharge from an intensive care unit (ICU) among survivors of critical illness. However, in contrast with our results, those authors did not find significant differences post-6 months between frail and non-frail groups for the physical component of HRQoL. This discrepancy between our and their findings may be related to low HRQoL scores post-6 months in Bagshaw et al., even in the disabled non-frail group due to their critical illness. Nevertheless, taken together, results from both studies suggest that using frailty measures in acute care (ICU, ED) could help identify older patients with poorer HRQoL so that appropriate home care and community services could be provided after ED discharge for optimal recovery.

One important finding of our study is that both the mental and physical components were worse in our frail participants in the months following their ED visit for minor fractures. Past cross-sectional and prospective studies [7, 8] in seniors revealed that the physical dimension of HRQoL was affected by minor fractures while the mental component remained stable after discharge. Identifying frail older adults may help to refer them to existing services tailored to their needs, which might improve not only their physical functioning but also their mental health.

Our study also showed that HRQoL measures did not vary between 3 and 6 months within levels of frailty, except for the vulnerable group. These results are consistent with the concepts of vulnerability and reversibility associated with early stages of frailty [34]. On the one hand, as PCS scores decreased for the vulnerable group between the two time points, it could be hypothesized that this group may not have the capacity to resist the potential adverse effects of minor fractures as effectively as their fitter counterparts. Since physical activity has been shown to have a positive impact on HRQoL in pre-frail older adults [35], targeting this specific sub-group will help to provide them with appropriate interventions to prevent further decline of their HRQoL physical component. On the other hand, as HRQoL mental scale scores increased for that group between the two time points, our results suggest that vulnerable older adults, compared to their frailest counterparts, can recruit enough social and emotional resources to improve their MCS despite their PCS decline. Our results thus point to the relevance of categorizing older adults according to different levels of frailty for the purpose of identifying those who may present lower mental and physical HRQoL following ED visits.

One important clinical implication of the present study is that it highlights the potential importance of screening for frailty in the ED, a place where this is rarely done [36]. Older people with minor fractures currently do not receive differential ED care. An easy-to-administer frailty measure such as the CSHA-CFS [36] could help ED clinicians identify those who may need more clinical attention. To stratify the level of HRQoL post-discharge may help to implement healthcare approaches tailored to the specific needs of the frailest patients; these approaches have shown promising results in other clinical settings with respect to quality of life outcomes [15, 16].

Our findings should be interpreted in light of several limitations. First, HRQoL scores may have been underestimated due to attrition bias (approximately 20 %) at 3 and 6 months. However, further analysis revealed no difference in dropout rates between the 3 groups at 3 and 6 months. Second, HRQoL was not measured during the ED phase. The lack of SF-12 measures at baseline raises the question of whether a change might have been observed in HRQoL from ED discharge up to 3 months and could not provide information on how HRQoL improved or deteriorated during this length of time. The first HRQoL measure may also have taken short after ED discharge (<6 weeks) to reduce delay, as one might expect recovery during this length of time. Third, no frailty measures were taken at 3 months and 6 months, which do not allow to known if a change in group classification (fit, vulnerable, frail) would have occurred over time. Fourth, the impact of some potential confounding variables on HRQoL scores, such as depression and anxiety, was not verified. However, the comorbidities that we controlled for in our study included items related to depression and anxiety. Fifth, frailty assessments could have been performed by 2 raters to increase reliability and prevent misclassification. However, the tool used showed high interrater reliability in a previous study [37]. Finally, the data should be interpreted with caution due to the small number of participants in the frailest group.

Current findings build on existing work. Results were obtained via a multicenter study consecutively recruiting a large sample of participants. Generalization of findings to the population of older adults visiting EDs for minor fractures is thus increased through these methodological strengths. The ability of the frailty measure to help identify older adults showing poorer HRQoL post-discharge has received little attention to date in specific clinical settings. To our knowledge, there have been no other studies conducted with independent older adults admitted to EDs for minor fractures, although this type of injury is frequent in non-disabled seniors [8, 38].

## Conclusion

This study demonstrates that older adults with minor fractures who are frail have poorer mental and physical HRQoL in the months following ED discharge, suggesting that they require special attention in clinical care. Our results thus support the importance of including some easy-to-administer frailty screening tools in routine ED assessments. Measuring the frailty status of older adults who suffered a minor fracture might improve clinical decision-making at discharge, such as offering them preventive strategies tailored to their needs, so that HRQoL could be improved in the following months. Future studies could document which services (e.g., referral to day hospital/center to improve physical function and increase social contacts) should be provided following ED visits to improve HRQoL observed in frail participants who have suffered a fracture.

## Ethics, consent and permissions

All patients provided written informed consent.

## Appendix

**Table 5:** Baseline characteristics of independent senior participants and dropouts at 3 and 6 months

	Participants	Dropouts	*p*-value*
	*N* = 334 (%)	*N* = 94 (%)	
Age (years)			
65–74	148 (44.3)	35 (37.2)	0.2
75–85	146 (43.7)	41 (43.6)	
85 +	40 (12.0)	18 (19.2)	
Gender			
Women	256 (76.9)	69 (73.4)	0.6
Men	77 (23.2)	24 (25.5)	
Schooling			
Primary	35 (10.7)	12 (12.9)	0.0008
Secondary	113 (34.6)	51 (54.8)	
College	66 (20.2)	15 (16.1)	
University	113 (34.6)	15 (16.1)	
Number of comorbidities			
0–4	198 (59.5)	59 (63.4)	0.5
5–13	135 (40.5)	34 (36.6)	
Living alone, without help	111 (33.3)	32 (34.4)	0.8
Living alone, with some help	124 (37.2)	38 (40.9)	0.5
Social Support Index (SSI > 63/100)	266 (80.8)	69 (77.5)	0.5
ED visits in the last 3 months	42 (12.8)	13 (14.0)	0.8
Falls in the last 3 months	62 (18.7)	13 (14.0)	0.3
Numbers of outings/week < 5	237 (73.8)	67 (72.0)	0.7
Occasional use of a walking aid (baseline)	59 (17.8)	19 (20.4)	0.6
Baseline BADL score (14/14)	312 (93.4)	87 (92.6)	0.8
Baseline IADL score (<14)	131 (39.2)	44 (46.8)	0.2
Injury mechanism^a^			
- Fall from own height	245 (74.7)	68 (72.3)	0.3
- Fall from greater height	44 (13.4)	13 (13.8)	
- Motor vehicle accident	8 (2.4)	0 (0.0)	
- Other	31 (9.5)	13 (13.8)	
Location of fracture (injured body region)^a^			
Head	18 (5.4)	7 (7.5)	0.2
Spine	11 (3.3)	4 (4.3)	
Upper limb	179 (53.6)	56 (59.6)	
Thorax	49 (14.7)	8 (8.5)	
Lower limb	66 (19.8)	19 (20.2)	
Multiple	11 (3.3)	0 (0.0)	
Description of injury			
- Fracture only	222 (66.5)	54 (57.4)	0.1
- Co-occurring injuries	112 (33.5)	40 (42.6)	
Injury Severity Score (ISS) (/75)			
1–2	35 (10.5)	11 (11.7)	0.7
3–4	238 (71.3)	63 (67.1)	
5 +	61 (18.3)	20 (21.3)	
Pain (Visual Analog Scale ≥ 7/10)	40 (12.1)	11 (12.0)	1.0
Short Falls Efficacy Scale-International (short FES-I) (<9.8)	143 (42.9)	48 (51.6)	0.3
Cognitive status (MoCA < 23/30 or TICS-modified ≤ 31/50)	88 (26.4)	23 (30.3)	0.5
CHSA-Frailty Scale			
Very Fit	72 (21.6)	21 (23.3)	0.4
Well	117 (35.0)	23 (25.6)	
Well + treated comorbidities	73 (21.9)	28 (31.1)	
Apparently vulnerable	35 (10.5)	7 (7.8)	
Mildly frail	28 (8.4)	8 (8.9)	
Moderately frail	9 (2.6)	3 (3.3)	

*Chi-Square ^a^ = Fisher test

## Abbreviations

CSHA-CFS: Canadian Study of Health and Aging--Clinical Frailty Scale
ED: emergency department
HRQoL: Health-related quality of life
MCS: Mental Component Summary scale
PCS: Physical Component Summary scale
RP: relative proportion
SF-12: Short Form-12 Health Survey

## References

[1] Albert M, McCaig LF, Ashman JJ (2013). Emergency department visits by persons aged 65 and over: United States, 2009–2010.

[2] Pines JM, Mullins PM, Cooper JK, Feng LB, Roth KE (2013). National trends in emergency department use, care patterns, and quality of care of older adults in the United States. J Am Geriatr Soc.

[3] Owens PL, Russo CA, Spector W, Mutter R (2009). Emergency department visits for injurious falls among the elderly, 2006. Healthcare Cost and Utilization Project (HCUP) Statistical Briefs [Internet].

[4] Wilber ST, Blanda M, Gerson LW, Allen KR (2010). Short-term functional decline and service use in older emergency department patients with blunt injuries. Acad Emerg Med.

[5] Peterman A, Rothrock N, Cella D. Evaluation of health-related quality of life (HRQL) in patients with a serious life-threatening illness. 2015. http://www.uptodate.com/contents/evaluation-of-health-related-quality-of-life-hrql-in-patients-with-a-serious-life-threatening-illness. Accessed 26 June 2015.

[6] Lehmann P, Simmons CA. Tools for strengths-based assessment and evaluation. Springer Publishing Company; 2013. 534 pages. https://books.google.ca/books/about/Tools_for_Strengths_Based_Assessment_and.html?id=Axd8rLFuUyIC&redir_esc=y). Accessed 26 June 2015

[7] Sanfélix-Genovés J, Hurtado I, Sanfélix-Gimeno G, Reig-Molla B, Peiró S (2011). Impact of osteoporosis and vertebral fractures on quality-of-life. a population-based study in Valencia, Spain (The FRAVO Study). Health Qual Life Outcomes.

[8] González N, Aguirre U, Orive M, Zabala J, García-Gutiérrez S, Las Hayas C (2014). Health-related quality of life and functionality in elderly men and women before and after a fall-related wrist fracture. Int J Clin Pract.

[9] Tom SE, Adachi JD, Anderson FA, Boonen S, Chapurlat RD, Compston JE (2013). GLOW Investigators. Frailty and fracture, disability, and falls: a multiple country study from the global longitudinal study of osteoporosis in women. J Am Geriatr Soc.

[10] Chang YW, Chen WL, Lin FG, Fang WH, Yen MY, Hsieh CC (2012). Frailty and its impact on health-related quality of life: a cross-sectional study on elder community-dwelling preventive health service users. PLoS One.

[11] Lin CC, Li CI, Chang CK, Liu CS, Lin CH, Meng NH (2011). Reduced health-related quality of life in elders with frailty: a cross-sectional study of community-dwelling elders in Taiwan. PLoS One.

[12] Masel MC, Ostir GV, Ottenbacher KJ (2010). Frailty, mortality, and health-related quality of life in older Mexican Americans. J Am Geriatr Soc.

[13] Bilotta C, Bowling A, Case A, Nicolini P, Mauri S, Castelli M (2010). Dimensions and correlates of quality of life according to frailty status: a cross-sectional study on community-dwelling older adults referred to an outpatient geriatric service in Italy. Health Qual Life Outcomes..

[14] Bagshaw SM, Stelfox HT, Johnson JA, McDermid RC, Rolfson DB, Tsuyuki RT (2015). Long-term association between frailty and health-related quality of life among survivors of critical illness: a prospective multicenter cohort study. Crit Care Med.

[15] Hernández C, Alonso A, Garcia-Aymerich J, Serra I, Marti D, Rodriguez-Roisin R (2015). Effectiveness of community-based integrated care in frail COPD patients: a randomised controlled trial. NPJ Prim Care Respir Med..

[16] Leung DY (2015). The effect of a virtual ward program on emergency services utilization and quality of life in frail elderly patients after discharge: a pilot study. Clin Interv Aging..

[17] Fillenbaum GG (1988). Multidimensional functional assessment of older adults: The Duke Older American Resources and Services Procedures.

[18] Rockwood K, Song X, MacKnight C, Bergman H, Hogan DB, McDowell I (2005). A global clinical measure of fitness and frailty in elderly people. CMAJ.

[19] Ware J, Kosinski M, Keller SD (1996). A 12-Item Short-Form Health Survey: construction of scales and preliminary tests of reliability and validity. Med Care.

[20] Resnick B, Nahm ES (2001). Reliability and validity testing of the revised 12-item Short-Form Health Survey in older adults. J Nurs Meas.

[21] Abbreviated Injury Scale (AIS) 2005. Illinois: Association for the Advancement of Automotive Medicine Publications; 2008.

[22] Copes WS, Champion HR, Sacco WJ (1988). The injury severity score revisited. J Trauma.

[23] Daoust R, Beaulieu P, Manzini C (2008). Estimation of pain intensity in emergency medicine: a validation study. Pain.

[24] Fillenbaum G (1978). Multidimensional functional assessment: The OARS Methodology - A manual.

[25] Haywood KL, Garratt AM, Fitzpatrick R (2005). Older people specific health status and quality of life: a structured review of self-assessed instruments. J Eval Clin Pract.

[26] Kempen GI, Yardley L, van Haastregt JC, Zijlstra GA, Beyer N, Hauer K (2008). The Short FES-I: a shortened version of the falls efficacy scale-international to assess fear of falling. Age Ageing..

[27] Nasreddine ZS, Phillips NA, Bedirian V, Charbonneau S, Whitehead V, Collin I (2005). The Montreal Cognitive Assessment, MoCA: A brief screening tool for mild cognitive impairment. J Am Geriatr Soc.

[28] de Jager CA, Budge MM, Clarke R (2003). Utility of TICS-M for the assessment of cognitive function in older adults. Int J Geriatr Psychiatry.

[29] Vercambre MN, Cuvelier H, Gayon YA (2010). Validation study of a French version of the modified telephone interview for cognitive status (F-TICS-m) in elderly women. Int J Geriatr Psychiatry.

[30] Audet N, Lemieux M, Cardin J (2001). Enquête sociale et de santé 1998-Cahier technique et méthodologique: Définitions et composition des indices.

[31] Adachi JD, Adami S, Gehlbach S, Anderson FA, Boonen S, Chapurlat RD (2010). Impact of prevalent fractures on quality of life: baseline results from the global longitudinal study of osteoporosis in women. Mayo Clin Proc.

[32] Sirois MJ, Emond M, Ouellet MC (2013). Cumulative incidence of functional decline after minor injuries in previously independent older Canadian individuals in the emergency department. J Am Geriatr Soc.

[33] Al Sayah F, Majumdar SR, Johnson JA. Association of Inadequate Health Literacy with Health Outcomes in Patients with Type 2 Diabetes and Depression: Secondary Analysis of a Controlled Trial. Can J Diabetes. 2015. doi: 10.1016/j.jcjd.2014.11.005. [Epub ahead of print]. 10.1016/j.jcjd.2014.11.005 25825150

[34] Lang PO, Michel JP, Zekry D (2009). Frailty syndrome: a transitional state in a dynamic process. Gerontology.

[35] Kwon J, Yoshida Y, Yoshida H, Kim H, Suzuki T, Lee Y (2015). Effects of a combined physical training and nutrition intervention on physical performance and health-related quality of life in prefrail older women living in the community: a randomized controlled trial. J Am Med Dir Assoc.

[36] Goldstein JP, Andrew MK, Travers A (2012). Frailty in older adults using pre-hospital care and the emergency department: a narrative review. Can Geriatr J.

[37] Grossman D, Rootenberg M, Perri G-A (2014). Enhancing communication in end‐of‐life care: a clinical tool translating between the Clinical Frailty Scale and the Palliative Performance Scale. J Am Geriatr Soc.

[38] Edwards BJ, Song J, Dunlop DD, Fink HA, Cauley JA (2010). Functional decline after incident wrist fractures--Study of Osteoporotic Fractures: prospective cohort study. BMJ..

