# Erratum to: Frail older adults with minor fractures show lower health-related quality of life (SF-12) scores up to six months following emergency department discharge

**DOI:** 10.1186/s12955-016-0464-0

**Published:** 2016-04-11

**Authors:** Véronique Provencher, Marie-Josée Sirois, Marcel Émond, Jeffrey J. Perry, Raoul Daoust, Jacques S. Lee, Lauren E. Griffith, Brice Lionel Batomen Kuimi, Litz Rony Despeignes, Laura Wilding, Vanessa Fillion, Nadine Allain-Boulé, Johan Lebon, On behalf of the Canadian Emergency Team Initiative on Mobility in Aging

**Affiliations:** Université de Sherbrooke, and Centre de recherche sur le vieillissement, Sherbrooke, QC Canada; Université Laval, and Centre de Recherche du CHU de Québec, Quebec, QC Canada; University of Ottawa, and Ottawa Hospital Research Institute, Ottawa, ON Canada; Université de Montréal, and Research Center, Hôpital du Sacré-Cœur de Montréal, Montréal, QC Canada; University of Toronto, and Sunnybrook Health Science Center, Toronto, ON Canada; McMaster University, Hamilton, ON Canada

Unfortunately, during production of this article [1], the author Vanessa Fillion was missed from the author list. The correct author list can be seen above and the original article has been updated to reflect this.
